# On the validity of *Epeorella* Ulmer, 1939 (Ephemeroptera, Heptageniidae) with general considerations on the Heptageniidae of the Sunda Islands

**DOI:** 10.3897/zookeys.445.8370

**Published:** 2014-10-13

**Authors:** Michel Sartori

**Affiliations:** 1 Zoologisches Museum und Biozentrum Grindel, Martin-Luther-King-Platz 3, D-20146 Hamburg, Germany; 2 Museum of Zoology, Palais de Rumine, Place Riponne 6, CH-1005 Lausanne, Switzerland

**Keywords:** *Epeorella borneonia*, *Darthus vadorus*, Borneo, lectotype, new synonym, new status, new combination

## Abstract

The type material of *Epeorella borneonia* Ulmer, 1939, the sole species of the genus *Epeorella* Ulmer, 1939 is reinvestigated and a lectotype (male imago) is designated. Based on several morphological structures, the synonymy with *Epeorus* Eaton, 1881 (Rhithrogeninae) is rejected. *Epeorella* stat. prop., known only at the winged stages, belongs to the subfamily Ecdyonurinae, and is a probable endemic of the island of Borneo. The newly erected genus *Darthus* Webb & McCafferty, 2007, also endemic to Borneo and only known by one species at the nymphal stage, is shown to be a junior subjective synonym of *Epeorella*. The new combination *Epeorella vadora* (Webb & McCafferty, 2007) is proposed for the species. The distribution of known heptageniid species from the Sunda Islands is discussed.

## Introduction

In his major work devoted to the Ephemeroptera of the Sunda Islands, Ulmer (1939) described three new genera in the family Heptageniidae. He gave them names which recalled names of those allied genera he assumed were closely related: *Compsoneuriella* Ulmer, 1939 and *Compsoneuria* Eaton, 1881; *Rhithrogeniella* Ulmer, 1939 and *Rhithrogena* Eaton, 1881; *Epeorella* Ulmer, 1939 and *Epeorus* Eaton, 1881.

The Afrotropical genus *Notonurus* Crass, 1947 was put in synonymy with *Compsoneuriella* (type species *Compsoneuriella thienemanni* Ulmer, 1939, known from winged and nymphal stages) by Gillies (1963; 1984), which in turn was put in synonymy with *Compsoneuria* (Braasch and Soldán 1986b; Webb et al. 2006). Recent studies, however, have shown the three genera constitute monophyletic clades supported by synapomorphies (Sartori 2014b; Vuataz et al. 2013).

The genus *Rhithrogeniella* (type species *Rhithrogeniella ornata* Ulmer, 1939, based on winged stages only) had an enigmatic position for a long time, until another species from Vietnam (*Rhithrogeniella tonkinensis* Soldán & Braasch, 1986) was described, with the first reference to the nymphal stage. Based on these descriptions, Wang and McCafferty (2004) indicated that *Rhithrogeniella* was a synonym of *Rhithrogena* and *Rhithrogeniella ornata* was a species of *Rhithrogena*; the species *Rhithrogeniella tonkinensis* was transferred to the genus *Ecdyonurus*. Recently, the nymph of *Rhithrogeniella ornata* was described for the first time, and the generic status of *Rhithrogeniella* revalidated (Sartori 2014a) as a member of Ecdyonurinae.

The monotypic genus *Epeorella* (type species *Epeorella borneonia* Ulmer, 1939, known only from the winged stages) was synonymized with *Epeorus* (Wang and McCafferty 2004) on the basis of similarities in several characters which will be discussed below.

The family Heptageniidae is now divided into three subfamilies which can be broadly characterized as following (Kluge 1989; Webb and McCafferty 2008):

- Rhithrogeninae: nymph with a row of setae on the ventral surface of maxillae, with dorsal process of the forefemora projected and narrower than the ventral process, some genera with vestigial paracercus; winged stages with the median depression of the mesothoracic furcasternum convergent anteriorly, and prosternum lacking transverse and longitudinal ridges.
- Heptageniinae: nymph with a row of setae on the ventral surface of maxillae, with forefemora without a dorsal projection; winged stages with the median depression of the mesothoracic furcasternum convergent anteriorly, and prosternum with distinct transverse and longitudinal ridges.
- Ecdyonurinae: nymph with scattered setae on the ventral side of maxillae; winged stages with the median depression of the mesothoracic furcasternum parallel sided or divergent anteriorly, and prosternum generally lacking transverse and longitudinal ridges.

This study concludes the re-investigation of Ulmer’s Heptageniidae from Southeast Asia deposited in the Zoologisches Museum of Hamburg University (ZMH) (Sartori 2014a; b; c; d). The type material of *Epeorella borneonia* Ulmer, 1939 is described, some morphological structures are clarified, the subfamily position is established and a new synonymy is proposed.

## Material and methods

The studied material is composed of three pinned specimens. The female imago was rehydrated in a solution of trisodic phosphate 0.35% and then put in alcohol. Pictures were taken with a Visionary Digital Passport II in ZMH, and figs were assembled in Adobe Photoshop CS6.

## Results

### Epeorella borneonia

Taxon classificationAnimaliaEphemeropteraHeptageniidae

Ulmer, 1939

Epeorella borneonia : Ulmer 1939: 579 (male and female imago).
Epeorus borneonia : Wang and McCafferty 2004: 21.

#### Material examined.

One male imago, one female imago, one female subimago, all bearing the following labels: 1) Type [typewriting on red label], 2) Borneo, Nanga Serawei, 12–18.11.1924 3) Sammelreise Prof. Dr. H. Winckler, ded. 1924–1925 4) Z.M.H. Hamburg 5) G. Ulmer det. 1942 Vers. 13.9.1927. This last label is confusing, and according to Prof. H. Strümpel (in litt.) it is a probable mistake.

The male imago was wrongly mentioned as holotype by Weidner (1962). This terminology cannot be accepted because the “holotype” has not been designated by Ulmer and cannot be ascertained by the presence of a single specimen (see also Recommendation 73F. Avoidance of assumption of holotype, ICZN 1999).

The male imago is accordingly designated as LECTOTYPE of the species *Epeorella borneonia* by present designation.

The three specimens have been adequately described by Ulmer (1939). Only significant morphological characters are mentioned here.

#### Male imago.

**Figures 1–6.**
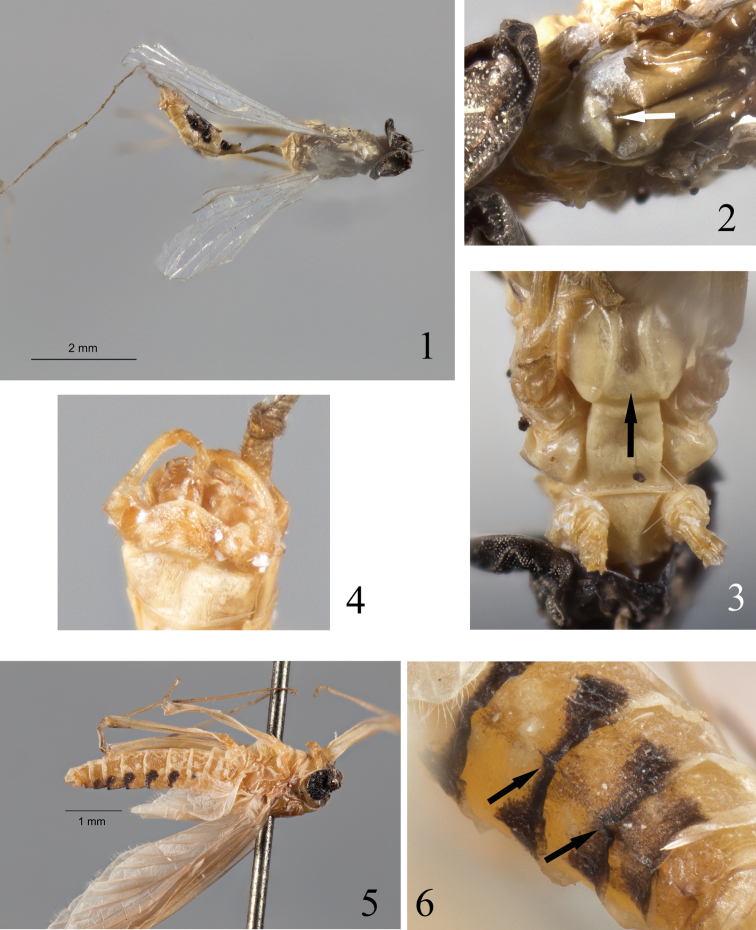
*Epeorella borneonia* Ulmer, 1939 **1** Lectotype male imago in dorsal view **2** Details of the mesonotum with transversal suture (arrow) **3** Detail of the mesothoracic furcasternum depression (arrow) **4** Detail of the genitalia in ventral view **5** Female subimago in lateral view **6** Detail of abdominal ridge (arrows) in dorsal view.

Anterior margin of the head not protruding anteriorly (Fig. 1); median depression of mesothoracic furcasternum subparallel, not convergent anteriorly (Fig. 3); mesonotum with a transverse suture (Fig. 2); styliger fig strongly convex, penis lobes minute, rounded and closely tight together (Fig. 4), without apparent sclerites or titillators [a complete analysis of the genitalia will be presented later with the help of non-invasive techniques].

#### Female imago.

Abdominal patterns similar to the male. Extracted eggs from the rehydrated specimen were unfortunately not in a satisfactory state for chorionic structure examination through SEM.

#### Female subimago.

Similar to the female, except abdominal terga VI–VIII (IV–V to a lesser extent) present the remains of a longitudinal ridge (Figs 5–6).

## Discussion

The synonymy of *Epeorella* with *Epeorus* was proposed by Wang and McCafferty (2004) based on the following assertions: i) male genitalia and forelegs are similar to those in *Epeorus*; ii) the vestiges associated with adults indicate that the larvae were two-tailed, and iii) the presence of median tubercles on abdominal segments VI–VIII can also be found in some *Epeorus* species.

The male genitalia greatly vary in shape among *Epeorus* species (see Webb and McCafferty 2008, figs 150–154) but are never as found in *Epeorella*; also, the forelegs of *Epeorella* are missing (see Ulmer 1939, p. 578) and therefore cannot be compared to *Epeorus*. The statement that, based on the vestigial paracercus, the nymph was two-tailed (hence comparable to *Epeorus*) is puzzling. “All Heptageniidae have the same vestigial paracercus, which does not allow to distinguish those with two-tailed and three-tailed larvae” (N. Kluge in litt.); the median ridge may be present on some *Epeorus* species which is true, but the genus *Epeorus* is absent from most of the Sunda Islands (Edmunds and Polhemus 1990, M. Sartori unpubl. data) as well as the Philippines. It is poorly diversified in Borneo where it is represented by a single species (*Epeorus boonsoongi* Braasch, 2011) with a large nymph of ca. 15 mm body length, and bifid tergal spines.

The male imago reinvestigated here presents all characteristics of the subfamily Ecdyonurinae, in peculiar the median depression of the mesothoracic furcasternum is not convergent anteriorly. Moreover, the presence of a clear transverse suture on the mesonotum, excludes it from the genus *Epeorus*. As already suggested by Braasch (2011) the synonymy proposed by Wang and McCafferty (2004) is incorrect and *Epeorella* is reinstated as *Epeorella* Ulmer, 1939, stat. prop.

**Figures 7–8.**
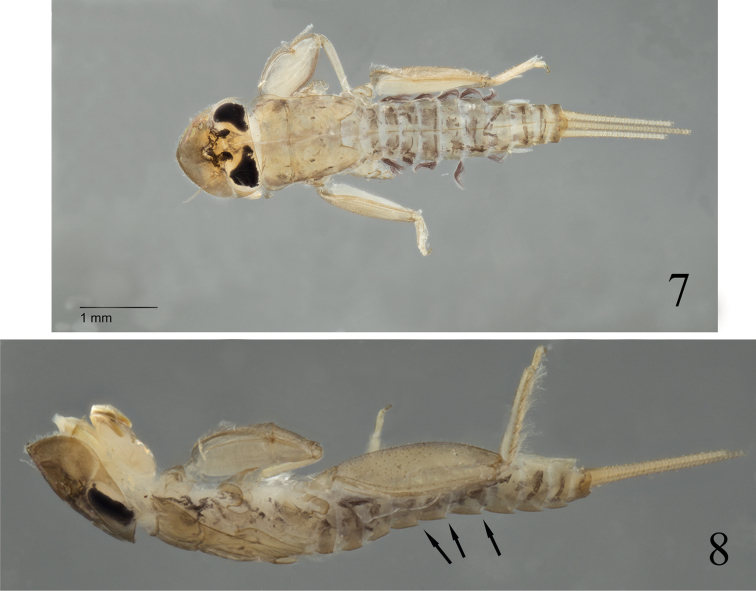
*Epeorella vadora* (Webb & McCafferty, 2007), comb. n. **7** Nymph paratype in dorsal view **8** Nymph paratype in lateral view with median abdominal ridge (arrows).

One interesting character of Wang and McCafferty (2004) is the presence of remains of median tubercles visible at least on abdominal terga VI–VIII of the female subimago. It has already been demonstrated that the subimaginal stage may retain some nymphal structures, such as gill sockets (Sartori et al. 2008), which may help to link nymphal and winged stages. The presence of vestigial tubercles on the terga thus indicates that the nymph possesses a median single ridge on the abdomen. Among Ecdyonurinae, two genera are known to hold such structures, *Notacanthurus* Tshernova 1974 known from East Palaearctic, the Himalaya region and Southeast Asia, and *Darthus* Webb and McCafferty 2007, only known from Borneo (Figs 7–8). Male imagos of *Notacanthurus* possess the anterior margin of the head distinctly produced (Braasch 1986; Webb and McCafferty 2008), and genitalia possess clearly visible apical and lateral sclerites. *Darthus* is only known from the nymphal stage from the same island as *Epeorella*. Although distant by ca 500 km, the type locality of *Epeorella borneonia* and that of *Darthus vadorus* Webb & McCafferty, 2007 belong to the Dipterocarpaceae forest of lowland altitudes (Sartori et al. 2003). Moreover, the size of the mature nymphs of *Darthus vadorus* (5.5–8.5 mm) is compatible with the adult size of *Epeorella borneonia* (5.0–5.5 mm) knowing that alate stages are generally smaller than mature nymphs. Therefore, it is likely that *Darthus* represents in fact the nymphal stage of *Epeorella*, and is considered as a subjective junior synonym of *Epeorella* **syn. n.** The species *Epeorella vadora* **comb. n.** is retained as a valid species because it exhibits a different colour pattern of the abdomen.

### General considerations on the Heptageniidae of the Sunda Islands

The three genera described by Ulmer (1939) have been put in synonymy by different authors but it is now demonstrated that they represent three groups of species deserving generic rank (Sartori 2014a; b, present study). They all belong to the subfamily Ecdyonurinae which is the most diversified in the studied area. Table 1 summarizes our current knowledge about the Heptageniidae of the Sunda Islands.

**Table 1.** Current species of the family Heptageniidae known from the Sunda Islands.

Subfamily	Genus	species	Sumatra	Java	Bali	Borneo	Lombok	Sumbawa	Sulawesi	Reference
Rhithrogeninae	*Rhithrogena*	*sumatrana* (Ulmer, 1939)	X	X			X			Sartori 2014d
	*Epeorus*	*boonsoongi* Braasch, 2011				X				Braasch 2011
Heptageniinae	*Trichogenia*	*nasuta* (Ulmer, 1939)	X							Webb et al. 2006
		*ulmeri* Braasch & Webb, 2006	X			X				Webb et al. 2006
		*hubleyi* Webb & McCafferty, 2006							X	Webb et al. 2006
Ecdyonurinae	*Afronurus*	*javanicus* Ulmer, 1939		X						Ulmer 1939
		*sarawakensis* Braasch, 2011				X				Braasch 2011
		*temburongensis* Braasch, 2005				X				Braasch 2005
		*webbi* Braasch, 2011				X				Braasch 2011
	*Asionurus*	*ulmeri* Braasch & Soldán, 1986	X	X						Braasch and Soldán 1986a
	*Atopopus*	*edmundsi* Wang & McCafferty, 1995				X				Wang and McCafferty 1995
		*tarsalis* Eaton, 1881				X				Sartori et al. 2007
	*Compsoneuria*	*lieftincki* (Ulmer, 1939)		X						Sartori 2014b
		*spectabilis* Eaton, 1881	X	X						Sartori 2014b
		sp.							X	Sartori 2014b
	*Compsoneuriella*	*thienemanni* Ulmer, 1939	X	X						Sartori 2014b
		sp.							X	Sartori 2014b
	*Epeorella*	*borneonia* Ulmer, 1939				X				Present study
		*vadora* (Webb & McCafferty, 2007)				X				Present study
	*Rhithrogeniella*	*ornata* Ulmer, 1939	X	X						Sartori 2014a
	*Thalerosphyrus*	*determinatus* (Walker, 1853)		X	X			X		Sartori 2014c
		*lamuriensis* Sartori, 2014	X							Sartori 2014c
		*sinuosus* (Navás, 1933)	X	X						Sartori 2014c
		sp.				X				Braasch 2011, Sartori 2014c
			9	9	1	10	1	1	3	

The Rhithrogeninae are known by only two species belonging to two widespread and speciose genera: *Rhithrogena* with more than 150 species and *Epeorus* with almost 100 species. Although mainly Holarctic, these two genera are present in the Oriental Region with 12 and 32 species respectively. The genus *Epeorus* is well represented in Indochina, where at least 13 species are known, but was unknown from the Sunda Islands until Braasch (2011) described *Epeorus boonsoongi* from Borneo. The genus is not recorded from Java, Sumatra and other Sunda Islands despite numerous samples over the last century. *Rhithrogena* exhibits an opposite trend in its distribution, being known from Sumatra, Java, Lombok, and possibly Bali, but seems to be absent from Borneo.

The only genus of the subfamily Heptageniinae present on the Sunda Islands is *Trichogenia* Braasch & Soldán, 1988, a Southeast Asian genus with one species in Sulawesi, *Trichogenia hubleyi* Webb & McCafferty, 2006 and two species in Sumatra, *Trichogenia nasuta* (Ulmer, 1939) and *Trichogenia ulmeri* Braasch & Webb, 2006, the latter also recorded from Borneo (Webb et al. 2006). There is a reasonable probability that *Trichogenia ulmeri*, known only at the nymphal stage, is a junior synonym of *Trichogenia nasuta*, known only at the winged stages, the distance between both type localities being less than 100 kilometres.

The subfamily Ecdyonurinae includes four times as many species as the two previous subfamilies combined. This is not surprising since Ecdyonurinae nymphs are among those which can tolerate slow flowing waters and high water temperatures; they have movable gills which is also an advantage when oxygen concentration is not optimal. The only two genera found in tropical Africa (*Afronurus* and *Notonurus*) also belong to the Ecdyonurinae. The genus *Afronurus* is the most diversified in the Oriental Region with 45 described species, but most of them are poorly known or badly described. It is probable that the concept of *Afronurus* in the Orient is paraphyletic; nevertheless, the genus seems present mainly on Borneo with three species; the species *Afronurus javanicus* Ulmer, 1939, is only known by adults collected on Java, which fit the current concept of *Afronurus* (M. Sartori, unpub. data). The genus is not reported from Sumatra, or Sulawesi, but seems present on Sumbawa and Sumba (M. Balke coll.). The two genera *Atopopus* and *Epeorella* are only found on Borneo, the former extending its range to the Philippines with two described species. The genera *Asionurus* and *Rhithrogeniella* have a distribution restricted to Indochina, extending to Java and Sumatra only. Finally Sulawesi is the most eastern island to have been colonized by Heptageniidae with *Compsoneuria*, *Compsoneuriella* and an undescribed genus (M. Sartori unpubl. data, M. Balke coll.). The family is not recorded from Moluccas, as well as Papua New Guinea, where the families Baetidae, Leptophlebiidae and Caenidae are eudominant.

More studies are needed, especially molecular phylogenies, to infer the timing and patterns of distribution of the genera and species in the area, particularly the relative importance of vicariance processes and dispersal events since the Miocene (Lohman et al. 2011).

## Supplementary Material

XML Treatment for Epeorella borneonia
